# Gonadal transcriptome sequencing of the critically endangered *Acipenser dabryanus* to discover candidate sex-related genes

**DOI:** 10.7717/peerj.5389

**Published:** 2018-07-27

**Authors:** Yeyu Chen, Ya Liu, Quan Gong, Jiansheng Lai, Mingjiang Song, Jun Du, Xiaochuan Deng

**Affiliations:** The Sichuan Academy of Agricultural Sciences, The Fishery Institute, Chengdu, China

**Keywords:** Transcriptome, Differentially expressed genes, Sex-related genes, *Acipenser dabryanus*, Ovary, Testis

## Abstract

**Background:**

*Acipenser dabryanus*, an endemic Chinese species, has been listed as a first-class protected animal in China. Sturgeons are among the oldest and most primitive group of existing fish in the world and occupy a special place in the evolutionary history of fish. Thus, a study of the reproduction and sex differentiation of sturgeon will be of great value for fish as well as the whole vertebrate group.

**Methods:**

In this study, we conducted comparative analysis of the testes and ovaries transcriptomes of *A. dabryanus* to screen for sex-differentiation and sexual development-related genes.

**Results:**

The transcriptome sequencing of six cDNA libraries generated 265 million clean reads, encompassing 79 Gb of sequences. The N50 and mean length of the identified 91,375 unigenes were 1,718 and 989 bp, respectively. A total of 6,306, 9,961, 13,170, 15,484, and 23,588 unigenes were annotated in the clusters of orthologous groups, gene ontology categories, Kyoto Encyclopedia of Genes and Genomes Pathway, euKaryotic orthologous groups, and NCBI non-redundant protein databases, respectively. A total of 5,396 differentially expressed genes were found between the two sexes, with 1,938 predicted to be up-regulated in ovaries and 3,458 in testes. A total of 73 candidate genes known to be involved in sex differentiation and sexual development were searched in the transcriptome of *A. dabryanus* of which 52 showed significant similarity. We highlighted six genes that are differentially expressed between the two sexes and may play important roles in sex differentiation and gonad maintenance. In addition, 24,271 simple sequence repeats (SSRs) and 550,519 single-nucleotide polymorphisms (SNPs) were detected.

**Discussion:**

This work represents the first transcriptome study comparing the ovary and testis in *A. dabryanus*. The putative differentially expressed genes between the gonads provide an important source of information for further study of the sex-differentiation related genes and the sex-differentiation mechanism in sturgeons. The SSRs or SNPs identified in this study will be helpful in the discovery of sex-related markers in *A. dabryanus*.

## Introduction

Sex is regarded as the queen of problems in evolutionary biology (Lewispye & Montalban, 2015). In the evolution of sex determination mechanisms in vertebrates, fish display diverse and complicated sex determination systems, and almost any sex determination mechanisms revealed in vertebrates has been discovered in fish (Pan et al., 2015). Therefore, the study of sex determination and differentiation in fish will help to reveal the formation and evolution of sex determination mechanisms of the whole vertebrate group. Sturgeons are among the oldest and most primitive group of existing fish in the world and are in the transition from cartilaginous fish to bony fish (Icardo et al., 2002). Therefore, considering the special evolutionary position of sturgeons, study on their reproduction and sex differentiation will be of great theoretical value for fish as well as vertebrates.

*Acipenser dabryanus*, also called Dabry’s sturgeon, is a freshwater sturgeon restricted to the Yangtze River system (Zen, 1990). The sexual maturity of *A. dabryanus* in males is reached at 4–6 years of age and in females at 6–8 years of age (Zhuang et al., 1997). Due to the heavy fishing, damming, and habitat degradation, the natural population of *A. dabryanus* has decreased dramatically in recent years and the wild species is almost extinct in the Yangtze River (Zhang et al., 2011; Li et al., 2015; Wu et al., 2014; Du et al., 2014). Consequently, *A. dabryanus* was listed as a first-class protected animal by the China Government (Wang, Yue & Chen, 1998) and also listed as a Critical Endangered species in the International Union for Conservation of Nature and Natural Resources Red List (www.iucnredlist.org/details/231/0). Thus, captive breeding of *A. dabryanus* may be the last chance for their survival and sustainability. However, it is very difficult to identify the sex of *A. dabryanus* based on secondary sexual characteristics during artificial propagation. Currently, growers usually wait 3–4 years before fish are sexed via an invasive surgical examination of the gonads (Doroshov, Moberg & Van Eenennaam, 1997). This limits the effective protection and artificial propagation of *A. dabryanus*. Therefore, a comprehensive study on the sex-differentiation and reproduction of *A. dabryanus* is crucial for their sustainable development.

The development and growth of sturgeons are very slow (Grandi & Chicca, 2008). Gonadal sex differentiation occurs late, and the histological differentiation time differs according to species. The earliest gonadal sex differentiation was observed at 3 months in *Acipenser gueldenstaedtii* (Akhundov & Fedorov, 1991), while most other sturgeons begin early gonad differentiation between 6 months and 9 months of age (Grandi, Giovannini & Chicca, 2007; Wrobel et al., 2002; Flynn & Benfey, 2007). Subsequently, it takes years for gonadal development to reach sexual maturity both in the wild and on fish farms. In theory, genes involved in sex differentiation might not function and express in the gonads of adult females or males but in immature individuals. In Chinese sturgeons, the closest species to Dabry’s sturgeon, the oocytes were still in the primary oocyte growth stage in 5-year-old females (Yue et al., 2015). Therefore, a comprehensive transcriptome study of the gonads in the immature period is essential to the discovery of the early sex-differentiation genes.

Transcriptome screening is one of the most powerful and efficient methods for discovering functional genes (Vidotto et al., 2013) as well as genetic markers. In the present study, we used the next-generation Illumina HiSeq platform to sequence the transcriptomes of *A. dabryanus.* This is the first transcriptome study to compare the ovary and testis in *A. dabryanus.* Our research can provide abundant information on the genes involved in reproduction and the general mechanism of gonad differentiation and development based on the gene expression profile. Moreover, the simple sequence repeats (SSRs) and single-nucleotide polymorphisms (SNPs) identified by transcriptome sequencing can contribute to the discovery of genetic or sex-linked markers in *A. dabryanus.*

## Materials and Methods

### Ethical procedures

All fish handling and experimental procedures were approved by the Animal Care and Use Committee of the Fishery Institute of the Sichuan Academy of Agricultural Sciences (20170226001A), and all animal collection and use protocols were carried out in accordance with the guidelines and regulations for the care and use of laboratory animals at the Fishery Institute of the Sichuan Academy of Agricultural Sciences.

### Sample materials

To discover potential sex-differentiation and sexual development-related genes, three developmental stages, including the early sex differentiation phase 9-month-old, 1-year-old, and 3-year-old immature individuals, were sampled separately at the Fishery Institute of the Sichuan Academy of Agricultural Sciences. One male and one female sturgeon were collected for transcriptome sequencing of each developmental stage. After anaesthetization by 0.05% MS-222 (Sigma, St. Louis, MO, USA), the testis and ovary tissues were collected and immediately flash frozen in liquid nitrogen, then transferred to an ultralow freezer at −80 °C until RNA extraction. Gonadal tissues (9 months old, 1 year old, and 3 years old) were fixed in Bouin’s solution, dehydrated by ethanol, cleared, and embedded in paraffin. Gonads were cut into eight-μm paraffin sections and stained with haematoxylin and eosin to identify the sex of Dabry’s sturgeon.

### RNA-Seq library preparation and sequencing

Total RNA was extracted from gonads using Trizol reagent (Invitrogen, Carlsbad, CA, USA) and examined on a 1% agarose gel. RNA was quality-controlled by using a NanoPhotometer^®^ spectrophotometer (Implen, Westlake Village, CA, USA) and quantified using a Qubit^®^ RNA Assay Kit in a Qubit^®^ 2.0 Fluorometer (Life Technologies, Carlsbad, CA, USA). The NEBNext^®^ Ultra^™^ Directional RNA Library Prep Kit for Illumina^®^ (Neb, Beverly, MA, USA) was used for library construction according to manufacturer’s instructions. Briefly, poly-A mRNA was purified from total RNA samples with Magnetic Oligo (dT) Beads. Fragmentation buffer was added to the purified mRNA to break them into a suitable size (about 200 bp). The short fragments of mRNA were then used for the first strand strands with random hexamer primers. DNA polymerase I, dNTPs, RNase H, and buffer were added to the first strand and the second strand of cDNA were then synthesized. After purification with AMPure XP beads, double-stranded cDNA were further added with the “A” base and connected with sequencing adapters. Qubit2.0 (Thermo, Waltham, MA, USA) and Agilent 2100 (Agilent, Santa Clara, CA, USA) were used to check the concentration and insert size of the final cDNA libraries to ensure the quality. A total of six libraries for gonads in different developmental stages across the immature phase were prepared and sequenced separately using the Illumina HiSeq 2000. The libraries were named T1 (the library of the 3-year-old male), T2 (the library of the 3-year-old female), T3 (the library of the 1-year-old male), T4 (the library of the 1-year-old female), T5 (the library of the 9-month-old male), and T6 (the library of the 9-month-old female).

### Transcriptome assembly

The raw reads were trimmed and quality controlled by the software WipeAadpter.pl (Biomarker Technologies Co., Ltd, Beijing, China) to remove reads with adaptors. Reads with more than 50% of bases having a *Q*-value ≤20 were filtered out by using the software Fas-tq_filter (Biomarker Technologies Co., Ltd, Beijing, China). To avoid missing short genes or those displaying low expression-levels, all clean reads were assembled together to perform the de novo assembly with program Trinity (Grabherr et al., 2011). Assembly completeness was estimated using the Bench-marking universal single-copy orthologs (BUSCO) analysis (Simão et al., 2015). The lineage dataset used in BUSCO is actinopterygii_odb9. The sequences assembled in Trinity were grouped together to conduct the annotation analysis. The reads of each individual were aligned to the assembled de novo transcriptome to obtain gene expression levels.

### Gene annotations

All assembled sequences were used for BLAST (Altschul et al., 1997) searches and annotation against the NCBI non-redundant (Nr) protein (Deng et al., 2006), Swiss-Prot (Apweiler et al., 2004), gene ontology (GO) (Ashburner et al., 2000), clusters of orthologous groups (COG) (Tatusov, Galperin & Natale, 2000), euKaryotic orthologous groups (KOG) (Koonin et al., 2004), eggNOG (Huerta-Cepas et al., 2015), and protein family (Pfam) (Finn et al., 2013) databases using an *e*-value of 1*e*−5, and the Kyoto Encyclopedia of Genes and Genomes (KEGG) orthology (Kanehisa et al., 2004) results were obtained by comparing with KEGG database using KOBAS2.0 (Xie et al., 2011). After predicting the amino acid sequence of unigenes, the software HMMER (Eddy, 1998) was used to compare them with Pfam database to get the annotation information of unigenes. The annotation tool TransDecoder was used to predict coding regions in the transcripts.

### Differential expression

Clean reads from female samples including all three developmental stages (T2, T4, and T6) were grouped together as immature females to perform the following differential expression analysis and so were the male samples (T1, T3, and T5). Bowtie (Langmead et al., 2009) was used to realign the original reads to the Trinity-assembled de novo transcriptome, and RSEM was used to obtain an abundance estimation for each sample (Li & Dewey, 2011). The expression profiles of the transcripts in different cDNA libraries were detected with the gene expression method of fragments per kilobase of transcript per million mapped reads (Trapnell et al., 2010). Differential expression analysis was performed using edgeR (Robinson, McCarthy & Smyth, 2010) in the OmicShare tools, a free online platform for data analysis (www.omicshare.com/tools). The default parameters of edgeR were used, and differential expression genes (DEGs) were selected according to log2 fold change ≥1 and *P*-value >0.05. Based on the gene expression level of the different samples, differentially expressed transcripts were annotated against the Nr protein, Swiss-Prot, GO, COG, KOG, eggNOG, Pfam, and KEGG databases to identify underrepresented and overrepresented terms in the male and female gonad tissues.

### Microsatellite and SNP discovery

The microsatellite regions from the sequence data were identified using the program MISA, available online (http://pgrc.ipk-gatersleben.de/misa/). Six types of SSRs were examined: mono-, di-, tri-, tetra-, penta- and hexa-nucleotide repeats, and the compound SSR. The minimum repeat for each type of SSRs was set as 10, 6, 5, 5, 5, and 5 for mono-, di-, tri-, tetra-, penta-, and hexa-nucleotide, respectively.

STAR software (Dobin et al., 2013) was used to align the reads and unigenes of each specimen, and GATK (McKenna et al., 2010) was performed during SNP calling and used to identify SNPs. The identification standards satisfied the following conditions: no more than three continuous single-base mismatches within 35 bp and an SNP quality greater than 2.0 after deep normalization of the sequence.

### Validation of transcriptomic data

To validate the expression profiles of the differentially expressed genes, eight putative genes were chosen and determined by real-time RT-PCR using the SYBR Green Real-time PCR Master Mix (Toyobo, Tokyo, Japan) and the IQ Multicolor Detection System (Bio-Rad, Hercules, CA, USA). Primers for real-time PCR are displayed in Table S1. To detected the most stably expressed housekeeping gene, β-*actin*, *Gapdh*, and *EF-1* were selected as candidate genes. Cycle threshold (Ct) values of all three genes were calculated using ovaries and testes of different developmental stages as template. The software geNorm (Vandesompele et al., 2002) was used to check the most suitable housekeeping gene for Dabry’s sturgeon. The real-time PCR was performed using a volume of 20 μl containing one μl cDNA template, 10 μl 2 × SYBR Green real-time PCR Master Mix, 0.5 μl of each target gene primer (10 μM), and eight μl water. The PCR cycling conditions were as follows: 5 min at 95 °C; then 40 cycles of 20 s at 95 °C, 20 s at 55 °C, and 30 s at 72 °C; finally, 70–95 °C with a ramp up of 0.5 °C per 10 s was performed to generate a melting curve. Each sample was performed and analyzed in triplicates. The 2^–ΔΔCT^ method was used to analyze the expression level of the differentially expressed genes (Livak & Schmittgen, 2001).

## Results

### Histological analysis

**Figure 1:**
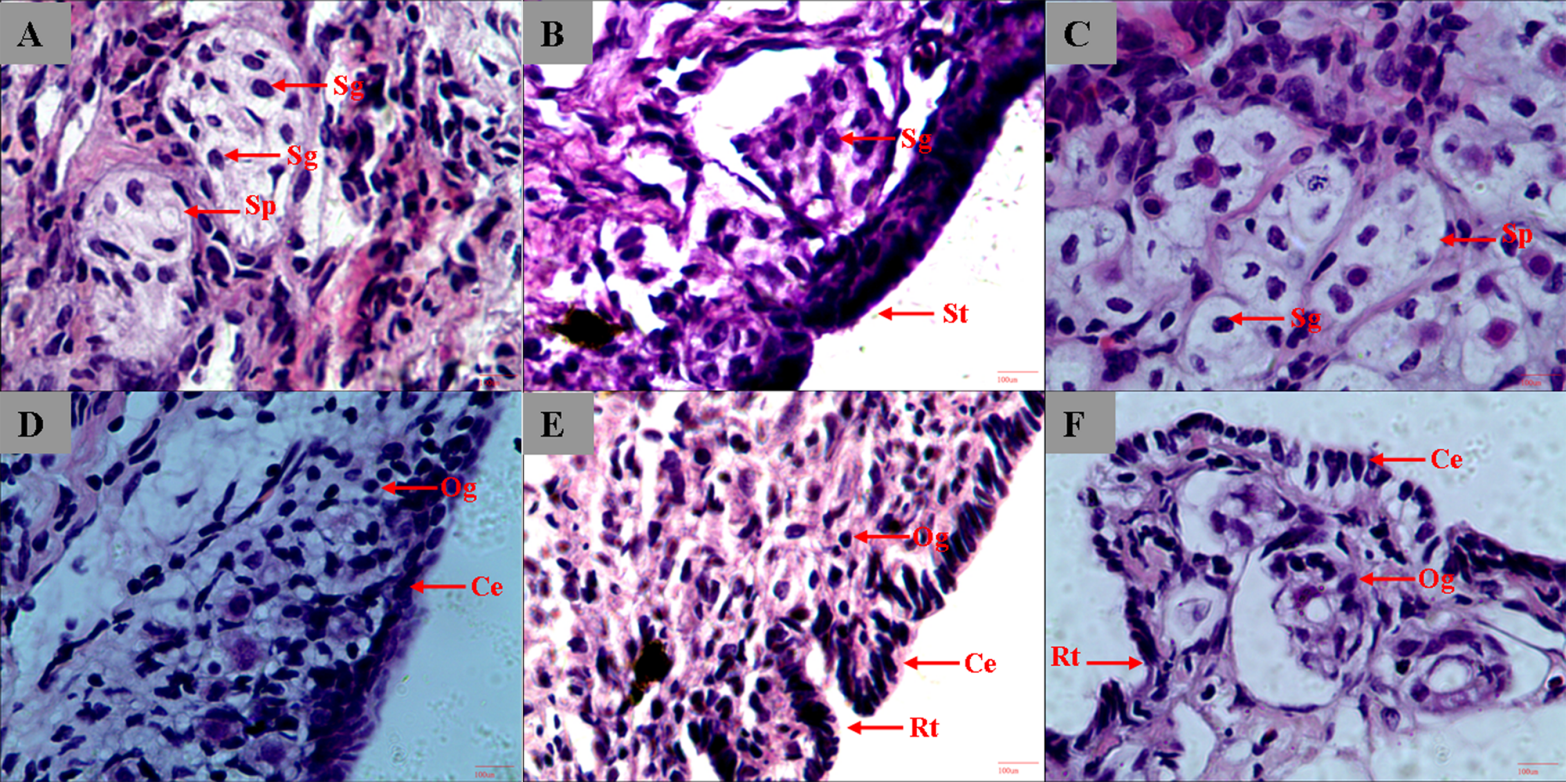
Histological section of *A. dabryanus* testis (A–C) and ovaries (D–F) at different developmental stages. (A) 9-month-old male; (B) 1-year-old male; (C) 3-year-old male; (D) 9-month-old female; (E) 1-year-old female; (F) 3-year-old female. Study sites: St, smooth type; Sp, spermatophore; Og: oogonium; Rt: ruga type; Sg: spermatogonia; Ce: cylindrical epithelium.

We started the sample collection of gonads 5 months from hatching, and the gonads remained undifferentiated at the histological level at 8 months. In 9-month-old sturgeon, gonads started to differentiate and spermatophore can be observed in testis and oogonium began to form in ovary (Figs. 1A and 1D). The different type of epithelium between ovary and testis was the most striking characteristic used to distinguish the male from female (Chen et al., 2004). In the 1-year-old and 3-year-old sturgeon, it is more obvious to see the ruga type and cylindrical epithelium in ovary and smooth type epithelium in testis (Figs. 1B, 1E and 1F). The connective tissue surround the spermatogonia and form into a circular or elongated spermatophore (Figs. 1A and 1C). The connective tissue and microvessels are rich in ovary tissue, the follicular shape is irregular and loose and oogonium can be observed inside the follicle (Figs. 1D, 1E and 1F).

### Sequencing and de novo assembly

Sequencing of the six cDNA libraries derived from the male and female gonads at 9 months, 1 year, and 3 years generated 265 million clean reads, encompassing 79 Gb of sequences. Transcriptome sequencing data have been deposited to the NCBI Sequence Read Archive database under accession numbers SRR6167299, SRR6172670, SRR6173479, SRR6175505, SRR6179331, and SRR6179394. The GC content frequency distribution of all reads ranged from 48.42% to 49.87%. The reads were assembled into 177,843 putative transcripts using Trinity with an N50 length of 1,908 bp and an average length of 1,210 bp. The N50 and mean length of the 91,375 unigenes were 1,718 and 989 bp, respectively (Table 1). Of the 91,375 unigenes, 63,271 unigenes (69.24%) were shorter than 1,000 bp, while 28,104 unigenes were longer than 1,000 bp long and 11,945 unigenes had a length of over 2,000 bp. BUSCO analysis showed that 3,872 genes (1,211 complete and single-copy BUSCOs, 1,987 complete and duplicated BUSCOs, 674 fragmented BUSCOs) were identified in the BUSCO database while the other 712 genes were the missing BUSCOs (Table 1). The unigene sequences were assigned with an ORF predictor TransDecoder, with a total of 48,623 unigenes (53.2%) detected putative CDS and according amino acid sequence.

**Table 1:** Summary of the *A. dabryanus* transcriptomes assembly.

Statistic	Transcript	Unigene
Total number	177,843	91,375
Total length	215,143,471	90,408,018
Average length	1,209.74	989.42
N50 length	1,908	1,718
Complete single-copy BUSCOs	1,211	
Complete duplicated BUSCOs	1,987	
Fragmented BUSCOs	674	
Missing BUSCOs	712	
Total BUSCO groups searched	4,584	
Total BUSCO genes recovered	3,872 (84%)	

### Annotation analysis

**Figure 2:**
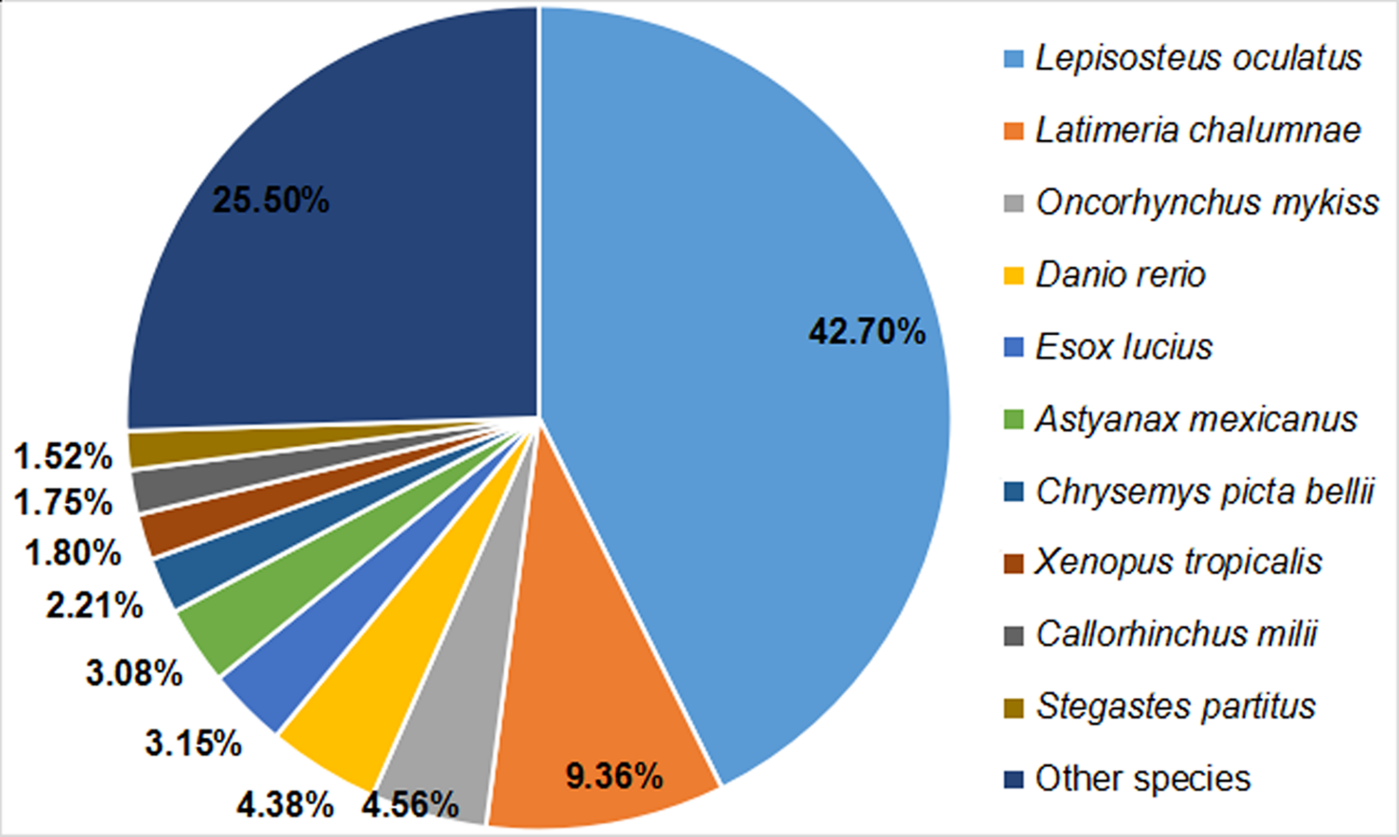
BLAST top-hit species distribution of homologous sequences in the *A. dabryanus* transcriptome. The number percentage of unigenes which are homologous with other species were presented.

Based on a comparison with protein sequences deposited in the Nr protein, Swiss-Prot, GO, COG, KOG, eggNOG, Pfam, and KEGG databases, we annotated a total of 24,978 unigenes within the Nr transcriptome of Dabry’s sturgeon. Significant matches were found for 6,306 unigenes in the COG database, 9,961 in the GO database, 13,170 in the KEGG database, 15,484 in the KOG database, 17,385 in the Pfam database, 13,515 in the Swiss-Prot database, 23,179 in the eggNOG database, and 23,588 in the Nr database (Table 2). Regarding homology with other species, 10,065 clusters (42.70%) were similar to *Lepisosteus oculatus*, followed by *Latimeria chalumnae*, *Oncorhynchus mykiss*, *Danio rerio*, *Esox lucius*, *Astyanax mexicanus*, *Chrysemys picta*, *Xenopus tropicalis*, *Callorhinchus milii*, *Stegastes partitus*, and other species (Fig. 2).

**Table 2:** Functional annotation of unigenes of the *A. dabryanus* transcriptome.

Database	Annotated number	300–1,000 (bp)	≥1,000 (bp)
COG	6,306	964	4,705
GO	9,961	2,009	7,616
KOG	15,484	3,020	11,708
Pfam	17,385	3,019	13,499
Swissprot	13,515	2,527	10,545
eggNOG	23,179	5,019	16,401
Nr	23,588	5,453	16,844
All annotated	24,978	5,759	17,020

**Figure 3:**
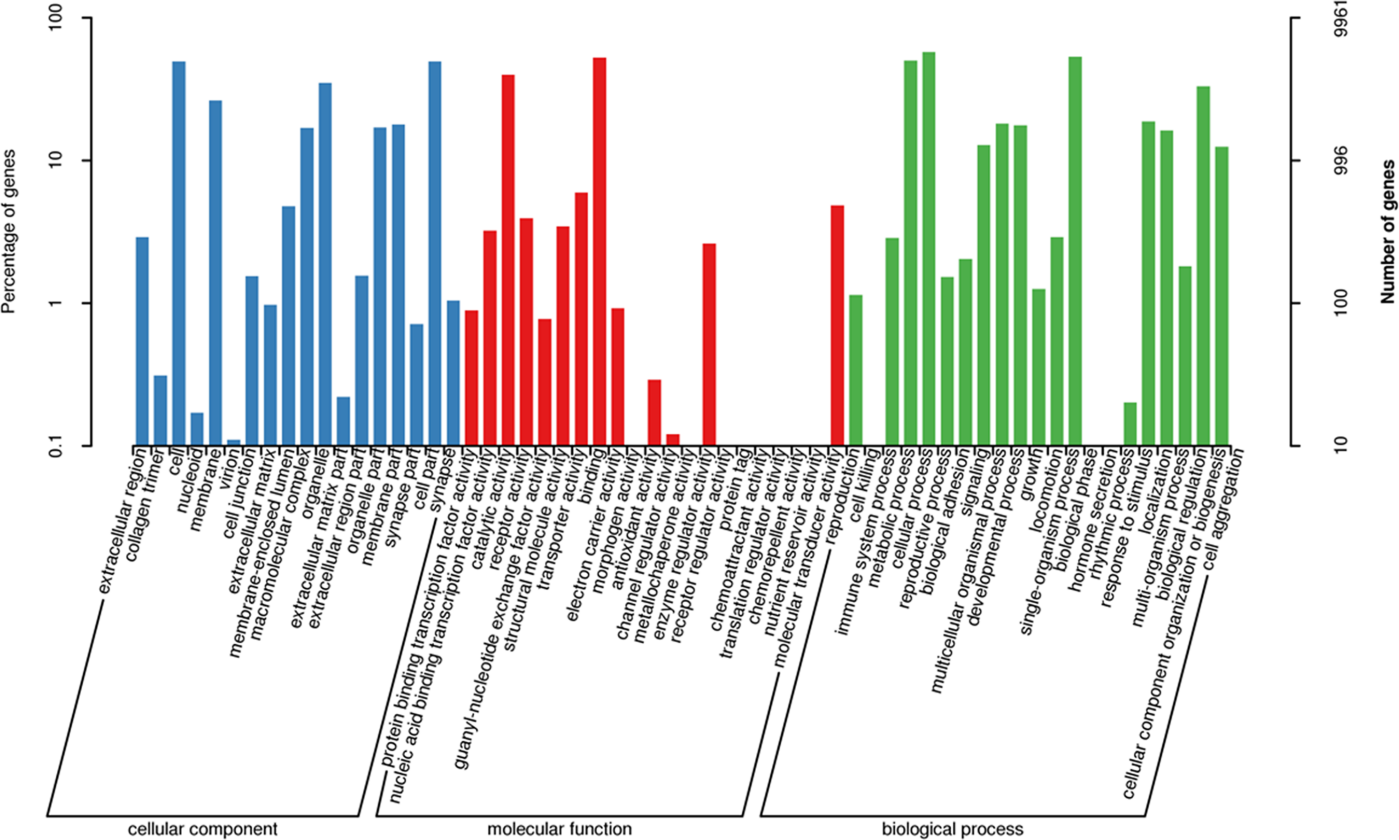
Functional gene ontology classification of the *A. dabryanus* gonadal transcriptome. Gene ontology analysis was performed for three main categories: cellular component, molecular function, and biological process.

The three main categories of GO annotations were 22,525 (34.79%) for cellular components, 11,925 (18.41%) for molecular function, and 30,303 (46.80%) for biological processes. In the cellular component ontology, the most abundant terms were cell, cell part, organelle, and membrane, whereas in the molecular function ontology, the most abundant terms were binding and catalytic activity; in the biological process ontology, we found that the most abundant terms annotated with the unigenes were cellular process, single-organism process, metabolism process, and biological regulation (Fig. 3).

**Figure 4:**
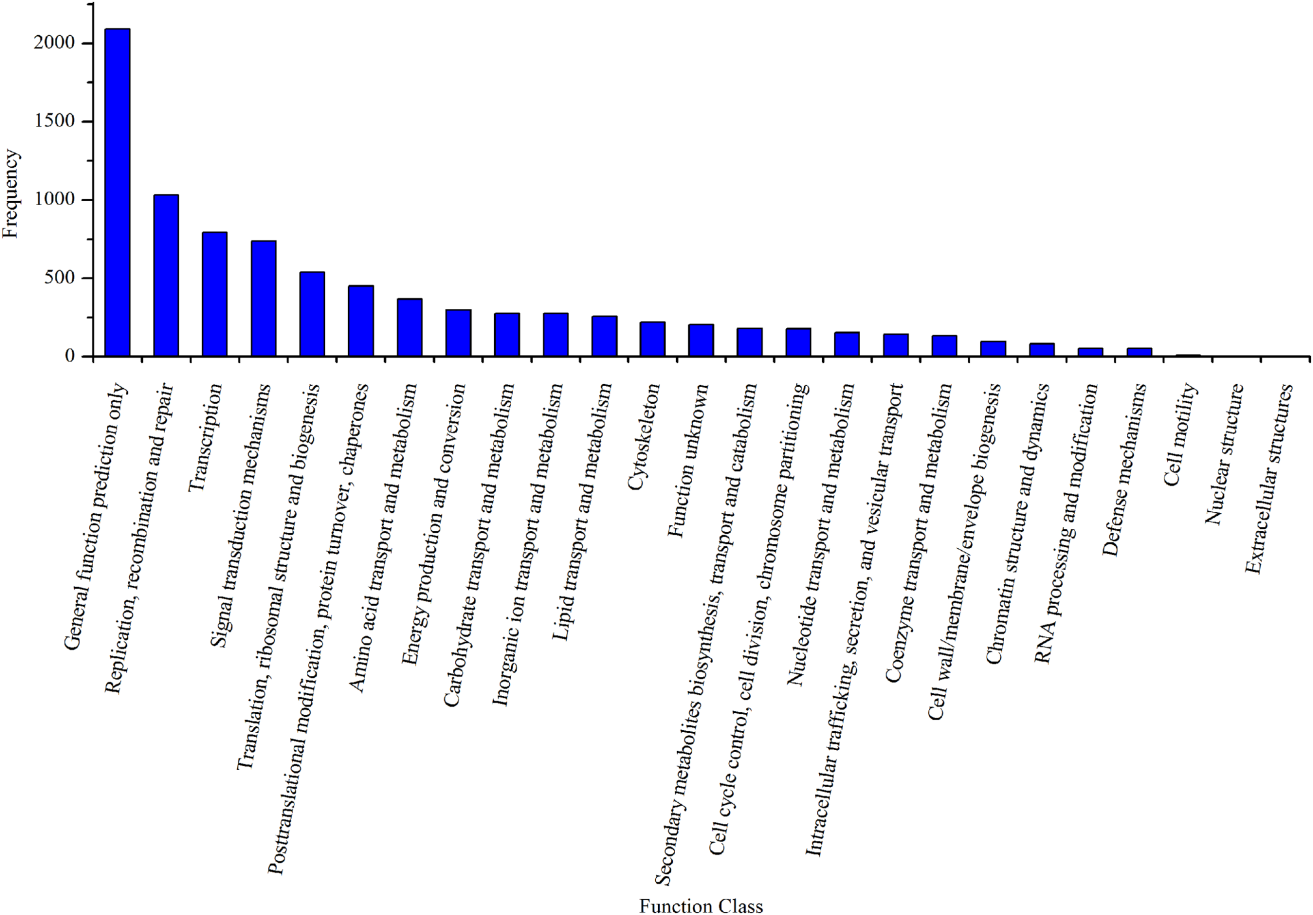
Clusters of orthologous group classifications of the *A. dabryanus* gonadal transcriptome. A total of 6,306 unigenes were grouped into 24 COG classifications and no unigene was found to belong to extracellular structures.

By mapping the unigenes to the COG database, we found that general function prediction only (2,092, 24.24%), replication, recombination and repair (1,033, 11.97%), translation (792, 9.18%), and signal transduction mechanisms (739, 8.56%) were the most frequently represented functional clusters in our transcriptome (Fig. 4). Only one unigene was annotated to the category nuclear structure, and no unigene was found to belong to extracellular structures.

Furthermore, unigenes were annotated in the KOG database and clustered into 25 KOG categories (Fig. S1), with “general function prediction only” containing the greatest number of unigenes (3,357, 19.28%), followed by “signal transduction mechanism” (3,007, 17.27%) and posttranslational modification, protein turnover, and chaperones (1,328, 7.63%).

We also mapped the unigenes to the KEGG pathway database and found that all the unigenes mapped to 290 pathways (Table S2). Briefly, of these sequences with a KEGG annotation, most genes (355, 2.47%) were from the MAPK signaling pathway, followed by focal adhesion (343, 2.39%), actin cytoskeleton regulation (304, 2.12%), endocytosis (292, 2.03%), the calcium signaling pathway (257, 1.79%), and ribosomes (256, 1.78%).

### Differentially expressed genes in different libraries and annotation analysis

**Figure 5:**
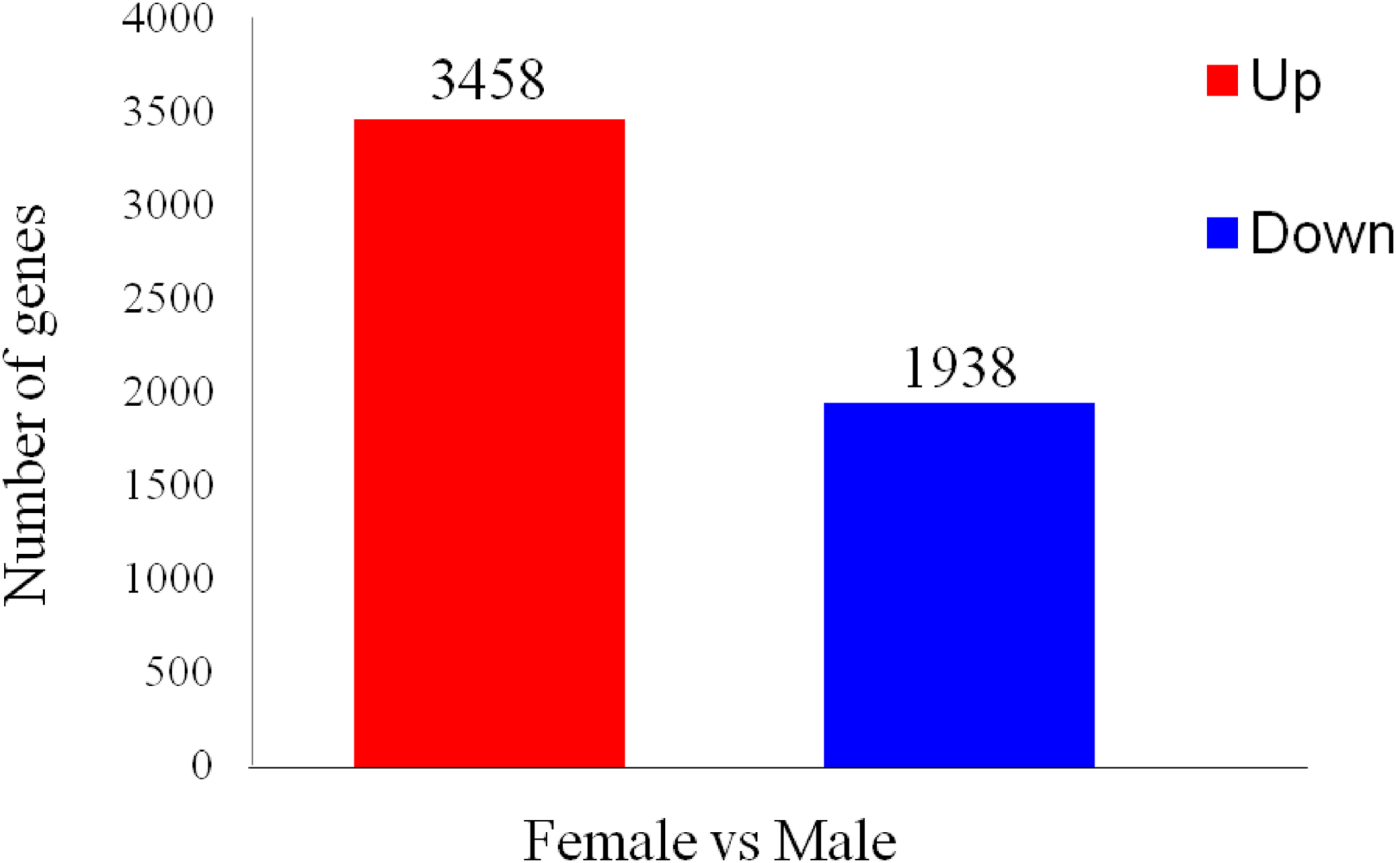
Differentially expressed gene (DEG) analysis of unigenes in the testes and ovaries of *A. dabryanus*. Female samples were used as control when comparing the up-regulated or down-regulated genes.

To identify differentially expressed transcripts in males and females, the ovaries or testes of three developmental phases were grouped together as immature females and males to be compared. A total of 5,396 differentially expressed genes were found between the two sexes, with 1,938 predicted to be up-regulated in ovaries and 3,458 in testes (Fig. 5).

All unigenes identified as differentially expressed were annotated. GO annotations revealed that the DEGs were mostly allocated to the following terms: molecular function, biological process, cellular component, single-organism process, and cellular process. All DEGs were annotated into 21 COG categories, with “general function prediction only” containing the greatest number of DEGs, followed by replication, recombination and repair, posttranslational modification, protein turnover, chaperones and amino acid transport, and metabolism. By mapping the DEGs to the KOG database, we found that over half of the DEGs were classified into the general function prediction only, signal transduction mechanism, cytoskeleton and posttranslational modification, protein turnover, and chaperone categories. In the KEGG pathway analysis, most DEGs between the two sexes were from pathways for cell adhesion molecules, neuroactive ligand-receptor interactions, MAPK signaling pathway, focal adhesion, and Wnt signaling pathway.

### Search for genes potentially involved in sex differentiation in Dabry’s sturgeon

We evaluated 73 candidate genes known to be involved in sex differentiation, sex determination, and sexual development to search for unigenes annotated in the transcriptome of Dabry’s sturgeon (Table S3). Significant matches were found for 52 of the 73 genes investigated (Table S3). Some genes shown to be sex-determining genes in other species, such as *Dmrt1*, *Fem1*, and *Gsdf*, were present in the gonad transcriptome of Dabry’s sturgeon. The anti-Müllerian hormone genes were missing in Dabry’s sturgeon. The SRY (sex-determining region Y)-box genes *Sox1*, *Sox3*, *Sox4*, *Sox6*, *Sox9*, *Sox11*, *Sox14*, and *Sox17*; steroidogenic enzymes *Cyp11a1*, *Cyp17a1*, *Cyp19a1a*, and *Fst*; and sex hormone receptors *Ar1*, *Ar2*, *Esr1*, *Esr2a*, and *Esr2b* were found in Dabry’s sturgeon. Other essential genes involved in sex differentiation and sexual development (*Foxl2*, *Bmp15*, *Gata4*, *Lhx1*, *Lhx9*, *Wnt*, *Nanos1*) were also detected.

### Identification of SNP and SSR markers

**Figure 6:**
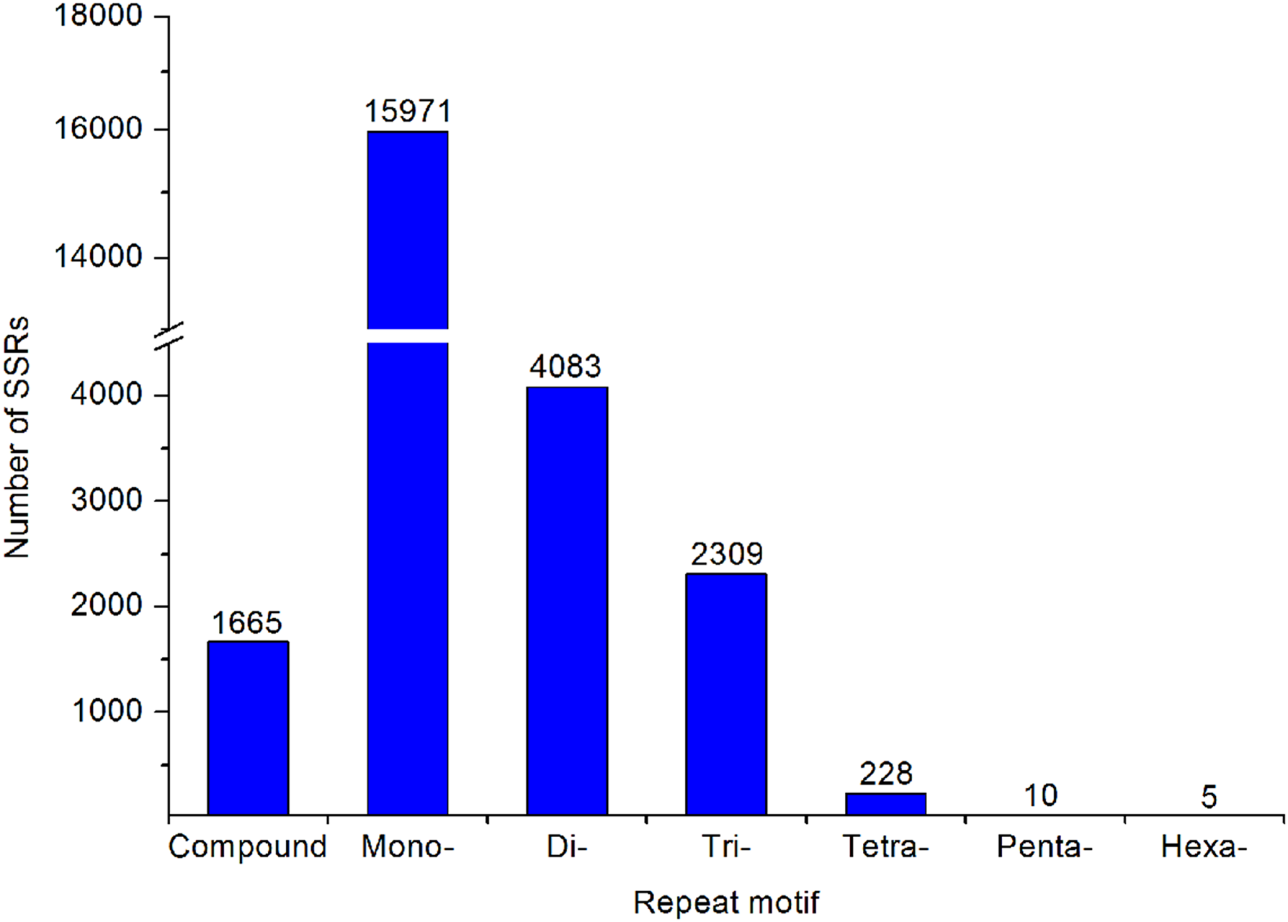
Summary of SSR types in the *A. dabryanus* transcriptome. The exact number of each SSR types was shown above the bars.

A large number of SSR and SNP markers were obtained. A total of 24,271 SSRs were identified from the transcriptomes of *A. dabryanus*. Disregarding mononucleotides, dinucleotides were the most abundant category, accounting for 61.53% of all SSRs, followed by trinucleotides (34.80%) and tetranucleotides (3.44%), as shown in Fig. 6. In contrast, penta (0.15%) and hexanucleotides (0.08%) were less abundant.

**Figure 7:**
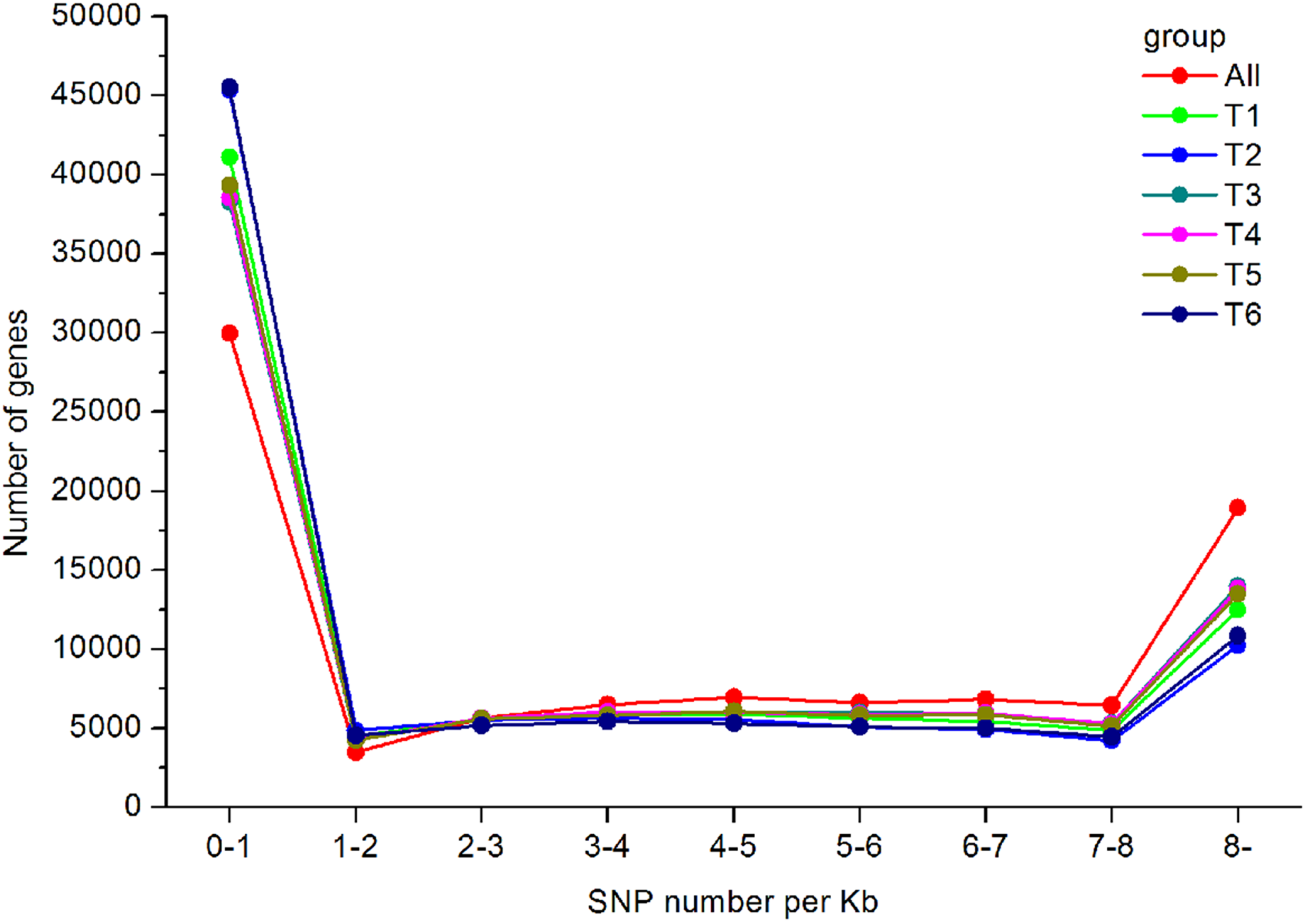
SNP distribution and density in the *A. dabryanus* transcriptome. The horizontal axis represented SNP numbers per Kilobase. The vertical axis represented the number of unigenes.

In total, 550,519 SNPs (453,779 from T1, 416,775 from T2, 485,960 from T3, 430,889 from T4, 489,734 from T5, and 480,501 from T6) were obtained. Regarding the type of SNPs, more heterozygous SNPs than homozygous SNPs were found in each library (Table 3). The distribution of the identified SNPs is shown in Fig. 7, and the majority of the unigenes only had one SNP.

**Table 3:** Statistics of SNP types in the *A. dabryanus* transcriptome.

Groups	HomoSNP	HeteSNP	All SNP
T1	201,415	252,364	453,779
T2	202,649	214,126	416,775
T3	197,180	288,780	485,960
T4	192,274	238,615	430,889
T5	196,165	293,569	489,734
T6	201,970	278,531	480,501

### Validation of gene expression by real-time PCR analysis

**Figure 8:**
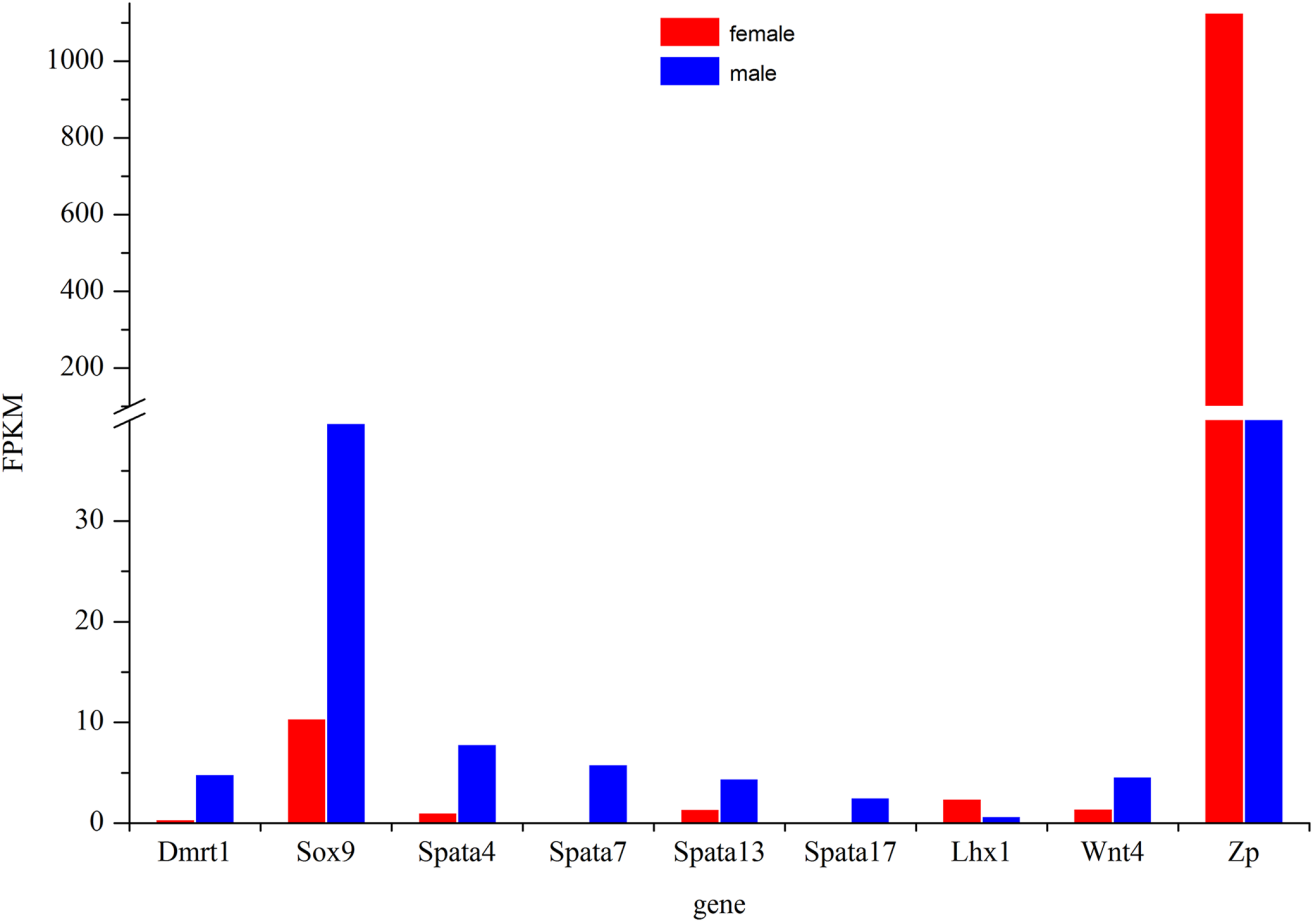
Expression profile of some major genes related to sex-differentiation or sexual development. The relative expression of these genes in females was shown in red and males was shown in blue.

The most commonly used reference genes β-*actin*, *Gapdh*, and *EF-1* were firstly amplified in different tissues of Dabry’s sturgeon to check the most stably expressed one. The average expression stability (*M* value) was calculated according to the Ct value of the housekeeping genes. Genes with the lowest *M* values have the most stable expression. In the present study, the *M* value for β-*actin*, *Gapdh*, and *EF-1* was 0.793, 1.36, and 0.861, respectively (Fig. S2). Therefore, β-*actin* was selected as the reference gene in Dabry’s sturgeon for the following qPCR analysis. To evaluate the transcriptome data, we chose eight differentially expressed genes (Fig. 8) potentially involved in sex differentiation, determination, or development for validation using real-time PCR. A comparison of the transcriptome data with the qPCR results revealed similar expression profiles, though the exact fold-change of these genes in different samples varied (Table 4). *Dmrt1* was also checked by real-time PCR with no expression level found in the 3-year old female gonad, so we failed to do the further statistics analysis for the real-time PCR.

**Table 4:** Comparison of relative expression of genes between RNA-seq and real-time PCR results.

Gene	FPKM (T/O)	qPCR(T/O)
*Wnt4*	3.18	2.58
*Lhx1*	0.29	0.16
*Zp*	0.09	0.25
*Spata4*	7.30	18.81
*Spata7*	56.74	80.63
*Sox9*	3.84	2.64
*Spata17*	28.19	38.50
*Spata13*	3.19	15.28
*Dmrt1*	12.14	–

**Notes:**

T/O, the relative expression level of genes in testis compared to that in ovary; T, testis; O, ovary.

“–” means there was no expression of the gene found in 3-year-old ovary.

## Discussion

### Transcriptome efficiency

Sturgeons are bony fish with remaining structural traits typical of the more primitive Chondrostei and are considered to be in the transitional position from Chondrostei to bony fish (Icardo et al., 2002). Thus, the study of the reproduction and sex-differentiation of sturgeons will enrich the research on evolutionary mechanism of the sex-determination and differentiation in fish. However, limited genetic information for Dabry’s sturgeon has hindered studies at the molecular level. This study was the first attempt at testicular and ovarian transcriptome sequencing in *A. dabryanus* using the Illumina HiSeq sequencing platform. The current study conducted deep sequencing of *A. dabryanus* encompassing over 10 Gb data for each library. In addition, we exclusively investigated gonads of immature phase to obtain information on the gene expression profile between the two genders. We used RNA-seq technology to identify a large number of unigenes annotated in various protein databases that can assist in the subsequent study of the gene expression profiling of *A. dabryanus*. After assembling, we obtained 91,375 unigenes with a mean length of 989 bp. The average length is much longer than that of the unigenes identified in previous studies (Chen et al., 2016; Hale, Jackson & Dewoody, 2010; Yue et al., 2015). Moreover, completeness analysis showed that our *A. dabryanus* transcriptome assembly contained 84% of the BUSCO core eukaryotic gene set, indicating the acceptable quality and high integrity of our assembled data. The large numbers of unigenes obtained in this study will be useful for future analyses of genes related to sex differentiation, reproduction, and marker detection in Dabry’s sturgeon.

### Genes related to reproduction

The *Dmrt1* gene is implicated in male determination and testis development in diverse metazoan phyla (Zhu et al., 2000; Hodgkin, 2002). In birds, which have ZW sex determination systems, previous studies have confirmed that *Dmrt1* was located on the Z chromosome but was absent from the W chromosome (Chue & Smith, 2011). However, the expression pattern of *Dmrt1* in fish is more complicated. In some species such as *Oryzias latipes* (Kobayashi et al., 2004), *Oreochromis niloticus* (Kobayashi et al., 2008), *Clarias gariepinus* (Raghuveer & Senthilkumaran, 2009), *Gobiocypris rarus* (Zhang, Zha & Wang, 2008), and *Paralichthys olivaceus* (Jo et al., 2007), *Dmrt1* was found to be specifically expressed in males, while in *D. rerio* (Guo et al., 2005), *O. mykiss* (Marchand et al., 2000), *Gadus morhua* (Johnsen et al., 2010), *Odontesthes bonariensis* (Fernandino et al., 2008), and *Silurus meridionalis* (Liu, Zhang & Wang, 2010), *Dmrt1* was found to be significantly up-regulated in males. Except for Adriatic sturgeon, which lacks the *Dmrt1* gene in both gonads (Vidotto et al., 2013), gonadal dimorphic *Dmrt1* expression has also been found in most sturgeons. The male-restricted and male-biased expression of *Dmrt1* was reported in Russian sturgeon (Chen et al., 2016) and lake sturgeon (Hale, Jackson & Dewoody, 2010), respectively, while no significant difference in its expression in the testes and ovaries was found in Amur sturgeon (Jin et al., 2015) and Chinese sturgeon (Yue et al., 2015). In the present study, *Dmrt1* expression was higher in males than in females (Fig. 8). The result indicated a strongly biased expression of *Dmrt1* in male Dabry’s sturgeons which is in accordance with the expression profile of *Dmrt1* in most other fish species.

The *Sox9* gene encodes a transcription factor that is critical for testis determination and development in vertebrates (Sinclair et al., 1990; Gubbay et al., 1990). Mutations in *Sox9* cause campomelic dysplasia, a human skeletal dysmorphology syndrome often associated with male to female sex reversal (Wagner et al., 1994; Foster et al., 1994). *Sox9* was also found to be expressed in fish and showed high expression levels in the testes compared with those in the ovaries in several species, such as *O. mykiss* (Takamatsu et al., 1997) and *Monopterus albus* (Zhou et al., 2003). Interestingly, the expression of *Sox9* in medaka was predominantly observed in the adult ovary, whereas in the testis, its expression was extremely low (Nakamoto et al., 2005; Yokoi et al., 2002), suggesting that the function of *Sox9* in the gonad is quite different in medaka and that the role of *Sox9* gene in sex-determination in fish is more complicated than in mammals. In sturgeons, the expression of the *Sox9* gene did not differ significantly in the ovary and testis in most of the studied species, such as Chinese sturgeon (Yue et al., 2015), lake sturgeon (Hale, Jackson & Dewoody, 2010) and Amur sturgeon (Jin et al., 2015). However, a recent study of *Sox9* in Russian sturgeon found that this gene was specifically expressed in males and revealed that the *Sox9* gene was responsible for the development of male gonads (Chen et al., 2016). In the present study, we found a significantly higher expression of *Sox9* in males than in females in the transcriptome of Dabry’s sturgeon (Fig. 8). The result suggested that, similar to *Dmrt1*, the *Sox9* gene might play a specific role in testis differentiation and maintenance in Dabry’s sturgeon.

The spermatogenesis-associated gene (*Spata*) was reported to be a candidate spermatocyte apoptosis-related gene involved in the regulation of apoptosis during spermatogenesis (Groh et al., 2013). *Spata4* was previous confirmed to be specifically expressed in testis ranging from mammals to birds (Liu et al., 2004; Xie et al., 2007). While in *D. rerio* and *O. mykiss*, the expression of *Spata4* is also restricted to gonads but with a slight expression level which can be detected in ovary (Liu et al., 2005a, 2005b). In the present study, *Spata4* showed a significantly higher expression level in males than females (Fig. 8), indicating that the function of this protein in sturgeon is in agreement with that in other fish. Besides *Spata4*, it is noticeable that all other genes belong to the Spata family demonstrated a male-bias expression profile, especially *Spata7* and *Spata17*, whose expression level in males was nearly 60-fold and 30-fold higher than that in females (Fig. 8; Table 4), suggesting a potential role of this gene during spermatogenesis in Dabry’s sturgeon.

Besides male-dominant expressed genes, we also found a significant up-regulated gene in the ovary named zona pellucida protein (*Zp*). *Zp* is a specialized extracellular matrix surrounding the developing oocyte and plays important roles in oocyte development and protection, fertilization, and spermatozoa binding (Hinsch et al., 1994; Gupta et al., 2012). In mice, the presence of an intact *Zp* could significantly improve the post-vitrification survival of oocytes compared to oocytes without *Zp* (Choi et al., 2015). In Dabry’s sturgeon, the expression of *Zp* in the ovary was over 10-fold higher than in the testis. The present result was consistent with the expression profile of *Zp* in Chinese sturgeon which found *Zp* was the primarily up-regulated gene in the ovary (Fig. 8). This finding implies that the *Zp* gene in Dabry’s sturgeon as well as other sturgeons may have similar functions to those found in other vertebrate and mammalian species, potentially indicating an essential role of *Zp* in oocyte maintenance.

*Fem1* is a sex-determining gene in *Caenorhabditis elegans* that functions in the signaling pathway that controls sex determination, and it is required in both germline and somatic tissues (Gaudet, VanderElst & Spence, 1996; Spence, Coulson & Hodgkin, 1990). *Fem1* has also been found in humans, mice, and zebrafish, and the sequence shows high conservation across vertebrates, implying its conserved role in developmental functions (Ventura-Holman et al., 2003). In the transcriptome of Dabry’s sturgeon, *Fem1* was identified but no differential expression was found between the sexes, consistent with previous studies in Amur sturgeon (Jin et al., 2015) and Chinese sturgeon (Yue et al., 2015) and suggesting that in sturgeons, *Fem1* may not play a key role in sex differentiation.

Other genes involved in the formation and maintenance of germ cells were also found in the transcriptome of Dabry’s sturgeon. In mice, the LIM homeobox gene is essential for mouse gonad formation, and a lack of *Lhx* expression will lead to a failure to form a discrete gonad (Birk et al., 2000). In Dabry’s sturgeon, we found LIM homeobox family genes *Lhx1* and *Lhx9*. The *Lhx1* gene was expressed significantly higher in females than in males (Fig. 8), while *Lhx9* was not differentially expressed between the two gonad types. *Lhx1* was found to be uniquely present in the testis transcriptome in Chinese sturgeon (Yue et al., 2015), contrary to the findings in the present study. Therefore, the accurate function of the LIM homeobox gene in sturgeons requires further investigation. The *Wnt* genes are sources of differentiation-inducing signals during normal developmental events and *Wnt4* has been clearly established to play a role in ovarian differentiation in mammals (Nusse & Varmus, 1992). In mice, the absence of *Wnt4* in female embryos results in the masculinization of XX gonads (Vainio et al., 1999). Compared with the specific role discovered in mammals, little is known regarding the role in gonad differentiation of *Wnt4* gene in fish. In a previous study, *Wnt4* was not detected in Chinese sturgeon while in Dabry’s sturgeon, *Wnt4* was identified in the ovary and testis with a significant sexual dimorphism in favor of male (Fig. 8). The male-dominant expression profile of *Wnt4* was also observed in some other fish such as *O. mykiss* (Nicol et al., 2012) and *Cynoglossus semilaevis* (Hu et al., 2014). The different expression profile of *Wnt4* between mammals and fish indicates that *Wnt4* might play diverse roles in development of the reproductive system.

### Identification of markers

Simple sequence repeats are tandem repeat DNA sequences that are highly polymorphic and are increasingly used as marker systems in molecular genetics studies, including research on parentage analysis (Poetsch et al., 2012), quantitative trait locus mapping (Keong et al., 2014), marker-assisted selection (Song et al., 2012) and population genetics (Remington et al., 2001). By transcriptome sequencing, we identified a large number of SSRs and analyzed the types and frequencies. In the transcriptome of Dabry’s sturgeon, dinucleotides were the most common microsatellites (accounting for up to 96.3%) if mononucleotide repeats were not taken into consideration, suggesting the existence of diverse dinucleotide repeat motif loci in Dabry’s sturgeon which are similar to those in other sturgeons such as Chinese sturgeon (Yue et al., 2015) and Russian sturgeon (Chen et al., 2016). Since Dabry’s sturgeon is a critically endangered species, SSRs identified from the transcriptome are useful markers for further assessment of genetic diversity and studies of population structure. Moreover, RAPD and AFLP have been applied in sturgeons to screen for sex-linked markers, but no sex-specific loci have been found (Wuertz et al., 2006; Keyvanshokooh, Pourkazemi & Kalbassi, 2007). Thus, the co-dominant microsatellite marker may be a more effective approach to search for sex-related markers in polyploid species.

## Conclusion

In the present study, we conducted a comparative analysis of gonadal transcriptomes in Dabry’s sturgeon. A total of 5,396 differentially expressed genes were found between both sexes, with 1,938 up-regulated in ovaries and 3,458 in testes. A total of 52 candidate genes known to be involved in sex differentiation, determination, and sexual development were found in the transcriptome of Dabry’s sturgeon. All genes belonging to the *Spata* family demonstrated a male-dominant expression profile, suggesting the crucial role of these genes during spermatogenesis in Dabry’s sturgeon. The male-biased expression of *Dmrt1* and *Sox9* in Dabry’s sturgeon also implied their potential role during testis development or differentiation. Similar to Chinese sturgeons, the expression of *Zp* in the ovary was significantly higher than in testis in Dabry’s sturgeon, indicating its potential conserved role in oocyte maintenance in vertebrate species. In addition, 24,271 SSRs and 550,519 SNPs were detected, which will assist further research on the discovery of sex-related markers.

## Supplemental Information

10.7717/peerj.5389/supp-1 Supplemental Information 1 Table S1. Primers for real-time PCR.

10.7717/peerj.5389/supp-2 Supplemental Information 2 Table S2. KEGG pathways found in the *A. dabryanus* transcriptome. 13,170 unigenes were mapped to 290 KEGG pathways.

10.7717/peerj.5389/supp-3 Supplemental Information 3 Table S3. Sex related genes used to search in *A. dabryanus* transcriptome. Significantly matched genes were highlighted in bold.

10.7717/peerj.5389/supp-4 Supplemental Information 4 Fig. S1. euKaryotic orthologous groups classification of *A. dabryanus* gonads transcriptome. 15,484 unigenes were grouped into 25 KOG classifications.

10.7717/peerj.5389/supp-5 Supplemental Information 5 Fig. S2. Average expression stability value (M) of three candidate reference genes. Gene expression stability of candidate reference genes in gonads was analyzed by the geNorm program. A lower value of average expression stability (M) indicates more stable expression.

## Additional Information and Declarations

### Data Availability

The following information was supplied regarding data availability: Transcriptome sequencing data have been deposited to the NCBI Sequence Read Archive (SRA) database under accession numbers SRR6167299, SRR6172670, SRR6173479, SRR6175505, SRR6179331, and SRR6179394.

## References

[ref-1] Akhundov MM, Fedorov KY (1991). Early gametogenesis and gonadogenesis in sturgeons. 1. On criteria for comparative assessment of juvenile gonadal development in the example of the Russian sturgeon, *Acipenser gueldenstaedtii*. Journal of Ichthyology.

[ref-2] Altschul SF, Madden TL, Schäffer AA, Zhang J, Zhang Z, Miller W, Lipman DJ (1997). Gapped BLAST and PSI BLAST: a new generation of protein database search programs. Nucleic Acids Research.

[ref-3] Apweiler R, Bairoch A, Wu CH, Barker WC, Boeckmann B, Ferro S, Gasteiger E, Huang HZ, Lopez R, Magrane M, Martin MJ, Natale DA, O’Donovan C, Redaschi N, Lai SL (2004). UniProt: the universal protein knowledgebase. Nucleic Acids Research.

[ref-4] Ashburner M, Ball CA, Blake JA, Botstein D, Butler H, Cherry JM, Davis AP, Dolinski K, Dwight SS, Eppig JT, Harris MA, Hill DP, Issel-Tarver L, Kasarskis A, Lewis S, Matese JC, Richardson JE, Ringwald M, Rubin GM, Sherlock G (2000). Gene ontology: tool for the unification of biology. Nature Genetics.

[ref-5] Birk OS, Casiano DE, Wassif CA, Cogliati T, Zhao L, Zhao Y, Grinberg A, Huang S, Kreidberg JA, Parker KL, Porter FD, Westphal H (2000). The LIM homeobox gene Lhx9 is essential for mouse gonad formation. Nature.

[ref-6] Chen XH, Wei QW, Yang DG, Zhu YJ, Liu Y (2004). Histological studies on gonadal origin and differentiation of cultured *Acipenser sinensis*. Journal of Fisheries of China.

[ref-7] Chen Y, Xia Y, Shao C, Han L, Chen X, Yu M, Sha Z (2016). Discovery and identification of candidate sex-related genes based on transcriptome sequencing of Russian sturgeon (*Acipenser gueldenstaedtii*) gonads. Physiological Genomics.

[ref-8] Choi JK, Yue T, Huang H, Zhao G, Zhang M, He X (2015). The crucial role of zona pellucida in cryopreservation of oocytes by vitrification. Cryobiology.

[ref-9] Chue J, Smith CA (2011). Sex determination and sexual differentiation in the avian model. FEBS Journal.

[ref-10] Deng YY, Li JQ, Wu SF, Zhu Y, Chen Y, He FC (2006). Integrated nr database in protein annotation system and its localization. Computer Engineering.

[ref-11] Dobin A, Davis CA, Schlesinger F, Drenkow J, Zaleski C, Jha S, Batut P, Chaisson M, Gingeras TR (2013). STAR: ultrafast universal RNA-seq aligner. Bioinformatics.

[ref-12] Doroshov SI, Moberg GP, Van Eenennaam JP (1997). Observations on the reproductive cycle of cultured white sturgeon, *Acipenser transmontanus*. Environmental Biology of Fishes.

[ref-13] Du H, Wei QW, Xie X, Shi LL, Wu JM, Qiao XM, Liu ZG (2014). Improving swimming capacity of juvenile Dabry’s sturgeon, (*Acipenser dabryanus*, Duméril, 1869) in current-enriched culture tanks. Journal of Applied Ichthyology.

[ref-14] Eddy SR (1998). Profile hidden Markov models. Bioinformatics.

[ref-15] Fernandino JI, Hattori RS, Shinoda T, Kimura H, Strobl-Mazzulla PH, Strüssmann CA, Somoza GM (2008). Dimorphic expression of dmrt1 and cyp19a1 (ovarian aromatase) during early gonadal development in pejerrey, *Odontesthes bonariensis*. Sexual Development.

[ref-16] Finn RD, Bateman A, Clements J, Coggill P, Eberhardt RY, Eddy SR, Heger A, Hetherington K, Holm L, Mistry J, Sonnhammer ELL, Tate J, Punta M (2013). Pfam: the protein families database. Nucleic Acids Research.

[ref-17] Flynn SR, Benfey TJ (2007). Sex differentiation and aspects of gametogenesis in shortnose sturgeon *Acipenser brevirostrum* Lesueur. Journal of Fish Biology.

[ref-18] Foster JW, Dominguez-Steglich MA, Guioli S, Kwok C, Weller PA, Stevanović M, Weissenbach J, Mansour S, Young ID, Goodfellow PN, Brook JD, Schafer AJ (1994). Campomelic dysplasia and autosomal sex reversal caused by mutations in an SRY-related gene. Nature.

[ref-19] Gaudet J, VanderElst I, Spence AM (1996). Post-transcriptional regulation of sex determination in *Caenorhabditis elegans*: widespread expression of the sex-determining gene fem-1 in both sexes. Journal of Molecular Cell Biology.

[ref-20] Grabherr MG, Haas BJ, Yassour M, Levin JZ, Thompson DA, Amit I, Adiconis X, Fan L, Raychowdhury R, Zeng Q, Chen Z, Mauceli E, Hacohen N, Gnirke A, Rhind N, di Palma F, Birren BW, Nusbaum C, Lindblad-Toh K, Friedman N, Regev A (2011). Full–length transcriptome assembly from RNA–Seq data without a reference genome. Nature Biotechnology.

[ref-21] Grandi G, Chicca M (2008). Histological and ultrastructural investigation of early gonad development and sex differentiation in Adriatic sturgeon (*Acipenser naccarii*, Acipenseriformes, Chondrostei). International Journal of Morphology.

[ref-22] Grandi G, Giovannini S, Chicca M (2007). Gonadogenesis in early developmental stages of *Acipenser naccarii* and influence of estrogen immersion on feminization. Journal of Applied Ichthyology.

[ref-23] Groh KJ, Schönenberger R, Eggen RIL, Segner H, Suter MJF (2013). Analysis of protein expression in zebrafish during gonad differentiation by targeted proteomics. General and Comparative Endocrinology.

[ref-24] Gubbay J, Collignon J, Koopman P, Capel B, Economou A, Münsterberg A, Vivian N, Goodfellow P, Lovell-Badge R (1990). A gene mapping to the sex-determining region of the mouse Y chromosome is a member of a novel family of embryonically expressed genes. Nature.

[ref-25] Guo Y, Cheng H, Huang X, Gao S, Yu H, Zhou R (2005). Gene structure, multiple alternative splicing, and expression in gonads of zebrafish Dmrt1. Biochemical and Biophysical Research Communications.

[ref-26] Gupta SK, Bhandari B, Shrestha A, Biswal BK, Palaniappan C, Malhotra SS, Gupta N (2012). Mammalian zona pellucida glycoproteins: structure and function during fertilization. Cell and Tissue Research.

[ref-27] Hale MC, Jackson JR, Dewoody JA (2010). Discovery and evaluation of candidate sex-determining genes and xenobiotics in the gonads of lake sturgeon (*Acipenser fulvescens*). Genetica.

[ref-28] Hinsch KD, Hinsch E, Meinecke B, Töpfer-Petersen E, Pfisterer S, Schill WB (1994). Identification of mouse ZP3 protein in mammalian oocytes with antisera against synthetic ZP3 peptides. Biology of Reproduction.

[ref-29] Hodgkin J (2002). The remarkable ubiquity of DM domain factors as regulators of sexual phenotype: ancestry or aptitude?. Genes & Development.

[ref-30] Hu Q, Zhu Y, Liu Y, Wang N, Chen S (2014). Cloning and characterization of wnt4a gene and evidence for positive selection in half-smooth tongue sole (*Cynoglossus semilaevis*). Scientific Reports.

[ref-31] Huerta-Cepas J, Szklarczyk D, Forslund K, Cook H, Heller D, Walter MC, Rattei T, Mende DR, Sunagawa S, Kuhn M, Jensen LJ, Von Mering C, Bork P (2015). eggNOG 4.5: a hierarchical orthology framework with improved functional annotations for eukaryotic, prokaryotic and viral sequences. Nucleic Acids Research.

[ref-32] Icardo JM, Colvee E, Cerra MC, Tota B (2002). Structure of the conus arteriosus of the sturgeon (*Acipenser naccari*i) heart. I: the conus valves and the subendocardium. Anatomical Record.

[ref-33] Jin SB, Zhang Y, Dong XL, Xi QK, Song D, Fu HT, Sun DJ (2015). Comparative transcriptome analysis of testes and ovaries for the discovery of novel genes from Amur sturgeon (*Acipenser schrenckii*). Genetics and Molecular Research.

[ref-34] Jo PG, An KW, Kim NN, Choi YA, Cho SH, Min BH, Han KL, Cheo YC (2007). Induced expression of doublesex-and mab-3-related transcription factor-1 (DMRT-1) mRNA by testosterone in the olive flounder, *Paralichthys olivaceus* ovary. Journal of Aquaculture.

[ref-35] Johnsen H, Seppola M, Torgersen JS, Delghandi M, Andersen O (2010). Sexually dimorphic expression of dmrt1 in immature and mature Atlantic cod (*Gadus morhua* L.). Comparative Biochemistry and Physiology Part B: Biochemistry and Molecular Biology.

[ref-36] Kanehisa M, Goto S, Kawashima S, Okuno Y, Hattori M (2004). The KEGG resource for deciphering the genome. Nucleic Acids Research.

[ref-37] Keong BP, Siraj SS, Daud SK, Panandam JM, Rahman ANA (2014). Identification of quantitative trait locus (QTL) linked to dorsal fin length from preliminary linkage map of molly fish, Poecilia sp. Gene.

[ref-38] Keyvanshokooh S, Pourkazemi M, Kalbassi MR (2007). The RAPD technique failed to identify sex-specific sequences in beluga (*Huso huso*). Journal of Applied Ichthyology.

[ref-39] Kobayashi T, Kajiura-Kobayashi H, Guan G, Nagahama Y (2008). Sexual dimorphic expression of DMRT1 and Sox9a during gonadal differentiation and hormone-induced sex reversal in the teleost fish Nile tilapia (*Oreochromis niloticus*). Developmental Dynamics.

[ref-40] Kobayashi T, Matsuda M, Kajiura-Kobayashi H, Suzuki A, Saito N, Nakamoto M, Shibata N, Nagahama Y (2004). Two DM domain genes, DMY and DMRT1, involved in testicular differentiation and development in the medaka, *Oryzias latipes*. Developmental Dynamics.

[ref-41] Koonin EV, Fedorova ND, Jackson JD, Jacobs AR, Krylov DM, Makarova KS, Mazumder R, Mekhedov SL, Nikolskaya AN, Rao BS, Rogozin IB, Smirnov S, Sorokin AV, Sverdlov AV, Vasudevan S, Wolf YI, Yin JJ, Natale DA (2004). A comprehensive evolutionary classification of proteins encoded in complete eukaryotic genomes. Genome Biology.

[ref-42] Langmead B, Trapnell C, Pop M, Salzberg SL (2009). Ultrafast and memory-efficient alignment of short DNA sequences to the human genome. Genome Biology.

[ref-43] Lewispye A, Montalban A (2015). A mathematical analysis of the evolutionary benefits of sexual reproduction. Journal of Organic Chemistry.

[ref-44] Li B, Dewey CN (2011). RSEM: accurate transcript quantification from RNA-Seq data with or without a reference genome. BMC Bioinformatics.

[ref-45] Li JX, Liu DQ, Ma QZ, Zhang XY, Dai W, Chen YB, Liu Y, Song ZB (2015). Discriminating Dabry’s sturgeon (*Acipenser dabryanus*) and Chinese sturgeon (*A. sinensis*) based on DNA barcode and six nuclear markers. Hydrobiologia.

[ref-46] Liu SF, He S, Liu BW, Zhao Y, Wang Z (2004). Cloning and characterization of testis-specific spermatogenesis associated gene homologous to human SPATA4 in rat. Biological & Pharmaceutical Bulletin.

[ref-47] Liu B, Liu S, He S, Zhao Y, Hu H, Wang Z (2005a). Cloning and expression analysis of gonadogenesis-associated gene SPATA4 from rainbow trout (*Oncorhynchus mykiss*). BMB Reports.

[ref-48] Liu S, Liu B, He S, Zhao Y, Wang Z (2005b). Cloning and characterization of zebra fish SPATA4, gene and analysis of its gonad specific expression. Biochemistry (Moscow).

[ref-49] Liu ZH, Zhang YG, Wang DS (2010). Studies on feminization, sex determination, and differentiation of the Southern catfish, *Silurus meridionalis*-a review. Fish Physiology and Biochemistry.

[ref-50] Livak KJ, Schmittgen TD (2001). Analysis of relative gene expression data using real time quantitative PCR and the 2^–ΔΔCT^ method. Methods.

[ref-51] Marchand O, Govoroun M, D’Cotta H, McMeel O, Lareyre JJ, Bernot A, Laudet V, Guiguen Y (2000). DMRT1 expression during gonadal differentiation and spermatogenesis in the rainbow trout, *Oncorhynchus mykiss*. Biochimica et Biophysica Acta.

[ref-52] McKenna A, Hanna M, Banks E, Sivachenko A, Cibulskis K, Kernytsky A, Garimella K, Altshuler D, Gabriel S, Daly M, DePristo MA (2010). The Genome Analysis Toolkit: a MapReduce framework for analyzing next-generation DNA sequencing data. Genome Research.

[ref-53] Nakamoto M, Suzuki A, Matsuda M, Nagahama Y, Shibata N (2005). Testicular type Sox9 is not involved in sex determination but might be in the development of testicular structures in the medaka, *Oryzias latipes*. Biochemical and Biophysical Research Communications.

[ref-54] Nicol B, Guerin A, Fostier A, Guiguen Y (2012). Ovary-predominant wnt4 expression during gonadal differentiation is not conserved in the rainbow trout (*Oncorhynchus mykiss*). Molecular Reproduction and Development.

[ref-55] Nusse R, Varmus HE (1992). Wnt genes. Cell.

[ref-56] Pan ZJ, Li XY, Zhou FJ, Qiang XG, Gui JF (2015). Identification of sex-specific markers reveals male heterogametic sex determination in *Pseudobagrus ussuriensis*. Marine Biotechnology.

[ref-57] Poetsch M, Bahnisch E, Ludescher F, Dammann P (2012). Maximising the power of discrimination is important in microsatellite-based paternity analysis in songbirds. Journal of Ornithology.

[ref-58] Raghuveer K, Senthilkumaran B (2009). Identification of multiple dmrt1s in catfish: localization, dimorphic expression pattern, changes during testicular cycle and after methyltestosterone treatment. Journal of Molecular Endocrinology.

[ref-59] Remington DL, Thornsberry JM, Matsuoka Y, Wilson LM, Whitt SR, Doebley J, Kresovich S, Goodman MM, Buckler ES (2001). Structure of linkage disequilibrium and phenotypic associations in the maize genome. Proceedings of the National Academy of Sciences of the United States of America.

[ref-60] Robinson MD, McCarthy DJ, Smyth GK (2010). edgeR: a Bioconductor package for differential expression analysis of digital gene expression data. Bioinformatics.

[ref-61] Simão FA, Waterhouse RM, Ioannidis P, Kriventseva EV, Zdobnov EM (2015). BUSCO: assessing genome assembly and annotation completeness with single-copy orthologs. Bioinformatics.

[ref-62] Sinclair AH, Berta P, Palmer MS, Hawkins JR, Griffiths BL, Smith MJ, Foster JW, Frischauf AM, Lovell-Badge R, Goodfellow PN (1990). A gene from the human sex-determining region encodes a protein with homology to a conserved DNA-binding motif. Nature.

[ref-63] Song W, Li Y, Zhao Y, Liu Y, Niu Y, Pang R, Miao G, Liao X, Shao C, Gao F, Chen S (2012). Construction of a high-density microsatellite genetic linkage map and mapping of sexual and growth-related traits in half–Smooth tongue sole (*Cynoglossus semilaevis*). PLOS ONE.

[ref-64] Spence AM, Coulson A, Hodgkin J (1990). The product of fem-1, a nematode sex-determining gene, contains a motif found in cell cycle control proteins and receptors for cell-cell interactions. Cell.

[ref-65] Takamatsu N, Kanda H, Ito M, Yamashita A, Yamashita S, Shiba T (1997). Rainbow trout SOX9: cDNA cloning, gene structure and expression. Gene.

[ref-66] Tatusov RL, Galperin MY, Natale DA (2000). The COG database: a tool for genome scale analysis of protein functions and evolution. Nucleic Acids Research.

[ref-67] Trapnell C, Williams BA, Pertea G, Mortazavi A, Kwan G, van Baren MJ, Salzberg SL, Wold BJ, Pachter L (2010). Transcript assembly and quantification by RNA Seq reveals unannotated transcripts and isoform switching during cell differentiation. Nature Biotechnology.

[ref-68] Vainio S, Heikkilä M, Kispert A, Chin N, McMahon AP (1999). Female development in mammals is regulated by Wnt-4 signalling. Nature.

[ref-69] Vandesompele J, Preter KD, Pattyn F, Poppe B, Roy NV, Paepe AD, Speleman F (2002). Accurate normalization of real-time quantitative RT-PCR data by geometric averaging of multiple internal control genes. Genome Biology.

[ref-70] Ventura-Holman T, Lu D, Si X, Izevbigie EB, Maher JF (2003). The Fem1c genes: conserved members of the Fem1 gene family in vertebrates. Gene.

[ref-71] Vidotto M, Grapputo A, Boscari E, Barbisan F, Coppe A, Grandi G, Kumar A, Congiu L (2013). Transcriptome sequencing and de novo annotation of the critically endangered Adriatic sturgeon. BMC Genomics.

[ref-72] Wagner T, Wirth J, Meyer J, Zabel B, Held M, Zimmer J, Pasantes J, Bricarelli FD, Keutel J, Hustert E, Wolf U, Tommerup N, Schempp W, Scherer G (1994). Autosomal sex reversal and campomelic dysplasia are caused by mutations in and around the SRY-related gene SOX9. Cell.

[ref-73] Wang S, Yue PQ, Chen YG (1998). China Red Data Book of Endangered Animals: Pisces.

[ref-74] Wrobel KH, Hees I, Schimmel M, Stauber E (2002). The genus Acipenser as a model system for vertebrate urogenital development: nephrostomial tubules and their significance for the origin of the gonad. Anatomy and Embryology.

[ref-75] Wu JM, Wei QW, Du H, Wang CY, Zhang H (2014). Initial evaluation of the release programme for Dabry’s sturgeon (*Acipenser dabryanus* Duméril, 1868) in the upper Yangtze River. Journal of Applied Ichthyology.

[ref-76] Wuertz S, Gaillard S, Barbisan F, Carle S, Congiu L, Forlani A, Aubert J, Kirschbaum F, Tosi E, Zane L, Grillasca JP (2006). Extensive screening of sturgeon genomes by random screening techniques revealed no sex-specific marker. Aquaculture.

[ref-77] Xie MC, Ai C, Jin XM, Liu SF, Tao SX, Li ZD, Wang Z (2007). Cloning and characterization of chicken SPATA4, gene and analysis of its specific expression. Molecular and Cellular Biochemistry.

[ref-78] Xie C, Mao X, Huang J, Ding Y, Wu J, Dong S, Kong L, Gao G, Li CY, Wei L (2011). KOBAS 2.0: a web server for annotation and identification of enriched pathways and diseases. Nucleic Acids Research.

[ref-79] Yokoi H, Kobayashi T, Tanaka M, Nagahama Y, Wakamatsu Y, Takeda H, Araki K, Morohashi KI, Ozato K (2002). Sox9 in a teleost fish, medaka (*Oryzias latipes*): evidence for diversified function of Sox9 in gonad differentiation. Molecular Reproduction and Development.

[ref-80] Yue HM, Li CJ, Du H, Zhang SH, Wei QW (2015). Sequencing and de novo assembly of the gonadal transcriptome of the endangered Chinese sturgeon (*Acipenser sinensis*). PLOS ONE.

[ref-81] Zen QZ (1990). Fisheries Resources in Yangtze Valley.

[ref-82] Zhang H, Wei QW, Du H, Li LX (2011). Present status and risk for extinction of the Dabry’s sturgeon (*Acipenser dabryanus*) in the Yangtze River watershed: a concern for intensified rehabilitation needs. Journal of Applied Ichthyology.

[ref-83] Zhang X, Zha J, Wang Z (2008). Influences of 4-nonylphenol on doublesex- and mab-3-related transcription factor 1 gene expression and vitellogenin mRNA induction of adult rare minnow (*Gobiocypris rarus*). Environmental Toxicology and Chemistry.

[ref-84] Zhou R, Liu L, Guo Y, Yu H, Cheng H, Huang X, Tiersch TR, Berta P (2003). Similar gene structure of two Sox9a genes and their expression patterns during gonadal differentiation in a teleost fish, rice field eel (*Monopterus albus*). Molecular Reproduction and Development.

[ref-85] Zhu L, Wilken J, Phillips NB, Narendra U, Chan G, Stratton SM, Kent SB, Weiss MA (2000). Sexual dimorphism in diverse metazoans is regulated by a novel class of intertwined zinc fingers. Genes & Development.

[ref-86] Zhuang P, Ke F, Wei QW, He XF, Chen YJ (1997). Biology and life history of Dabry’s sturgeon, *Acipenser dabryanus*, in the Yangtze River. Developments in Environmental Biology of Fishes.

