# Supplementary material for: Gonadal transcriptome sequencing of the critically endangered Acipenser dabryanus to discover candidate sex-related genes
Source: PeerJ. 2018 Jul 27;6:e5389. doi: 10.7717/peerj.5389 (PMC6065465; doi:10.7717/peerj.5389)
Supplement: Supplemental Information 1 [file peerj-06-5389-s001.doc]

| **Primers** | **Sequences** |
| --- | --- |
| *β-actin*-F | 5'-GACCGAGGCACCCCTGAAC-3' |
| *β-actin*-R | 5'-GATGGGCACTGTGTGTGTGAC-3' |
| *Dmrt1*-F | 5'-TGAACACGGTCACGGAGAGCTAT-3' |
| *Dmrt1*-R | 5'-ATGACTCGCTGTCGTTCTGCAATC-3' |
| *Wnt4*-F | 5'- ggaaagtggtggagatgcac-3' |
| *Wnt4*-R | 5'-aattcccctcaaagccgtcc-3' |
| *Sox9*-F | 5'-ATGCGGTTTCCCAGGTGCTG-3' |
| *Sox9*-R | 5'-CTTCATTCTGCCCGTTCTTCAC-3' |
| *Lhx1*-F | 5'-tgttggtttgatgacccagc-3' |
| *Lhx1*-R | 5'-gcccaaacaatatgccagag-3' |
| *Zp*-F | 5'-gtacagacatggtattcgtccg-3' |
| *Zp*-R | 5'-taccttcaagttccacccacc-3' |
| *Spata4*-F | 5'-tcaacctccacaaggaagtg-3' |
| *Spata4*-R | 5'-gctctctcaaactgcctctg-3' |
| *Spata7*-F | 5'-ccttccccagaggcatacag-3' |
| *Spata7*-R | 5'-ccactgtaggttttgttgtgag-3' |
| *Spata13*-F | 5'-CACAGCAGAGCCTGAACGCT-3' |
| *Spata13*-R | 5'-cgaggaggaggtcctcgat-3' |
| *Spata17*-F | 5'-cctctctgaagtgtttggtg-3' |
| *Spata17*-R | 5'-atctcggtcaagaaagcatgg-3' |
