# Supplementary figures and images for: Gonadal transcriptome sequencing of the critically endangered Acipenser dabryanus to discover candidate sex-related genes
Source: PeerJ. 2018 Jul 27;6:e5389. doi: 10.7717/peerj.5389 (PMC6065465; doi:10.7717/peerj.5389)

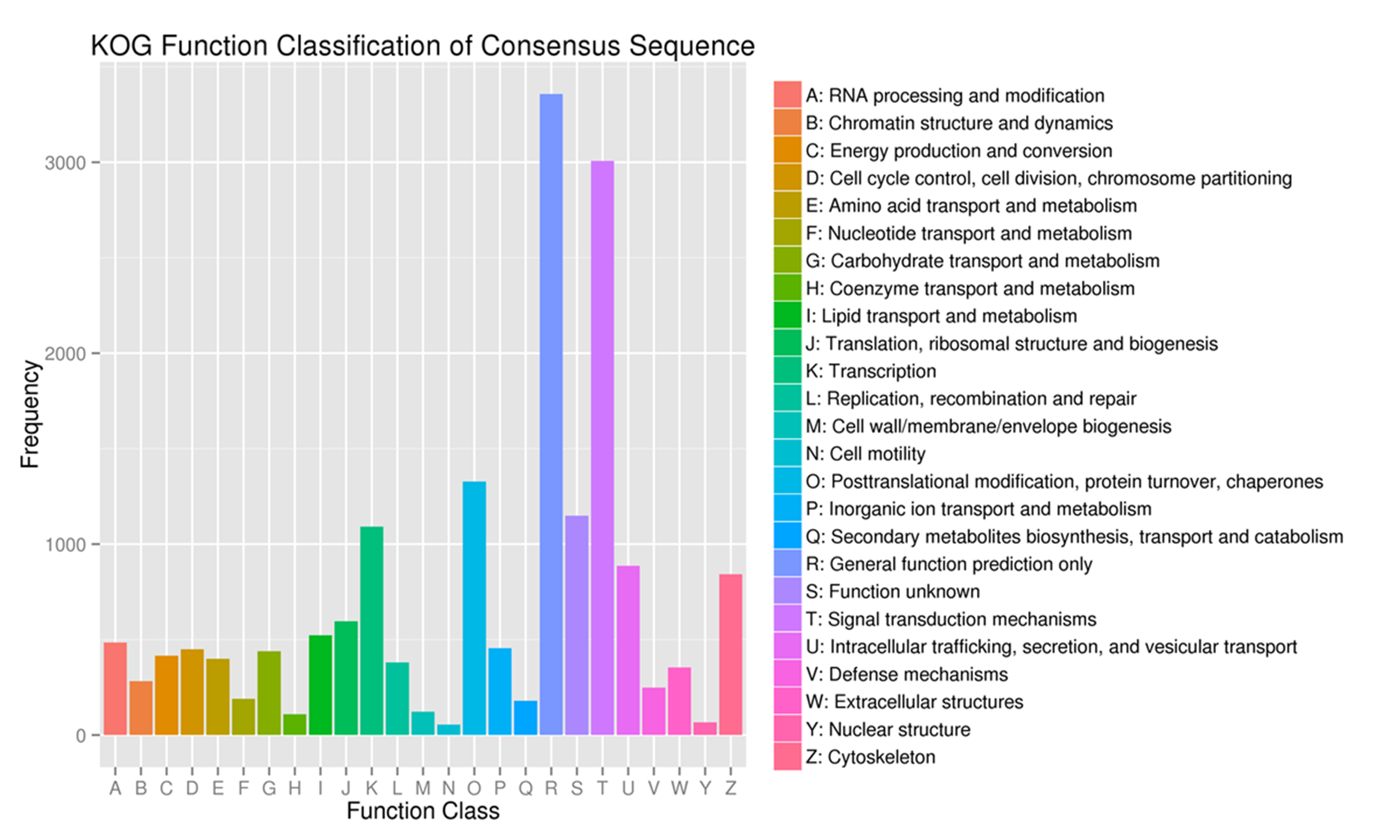

Supplement: Supplemental Information 4 — 15,484 unigenes were grouped into 25 KOG classifications. [file peerj-06-5389-s004.png]

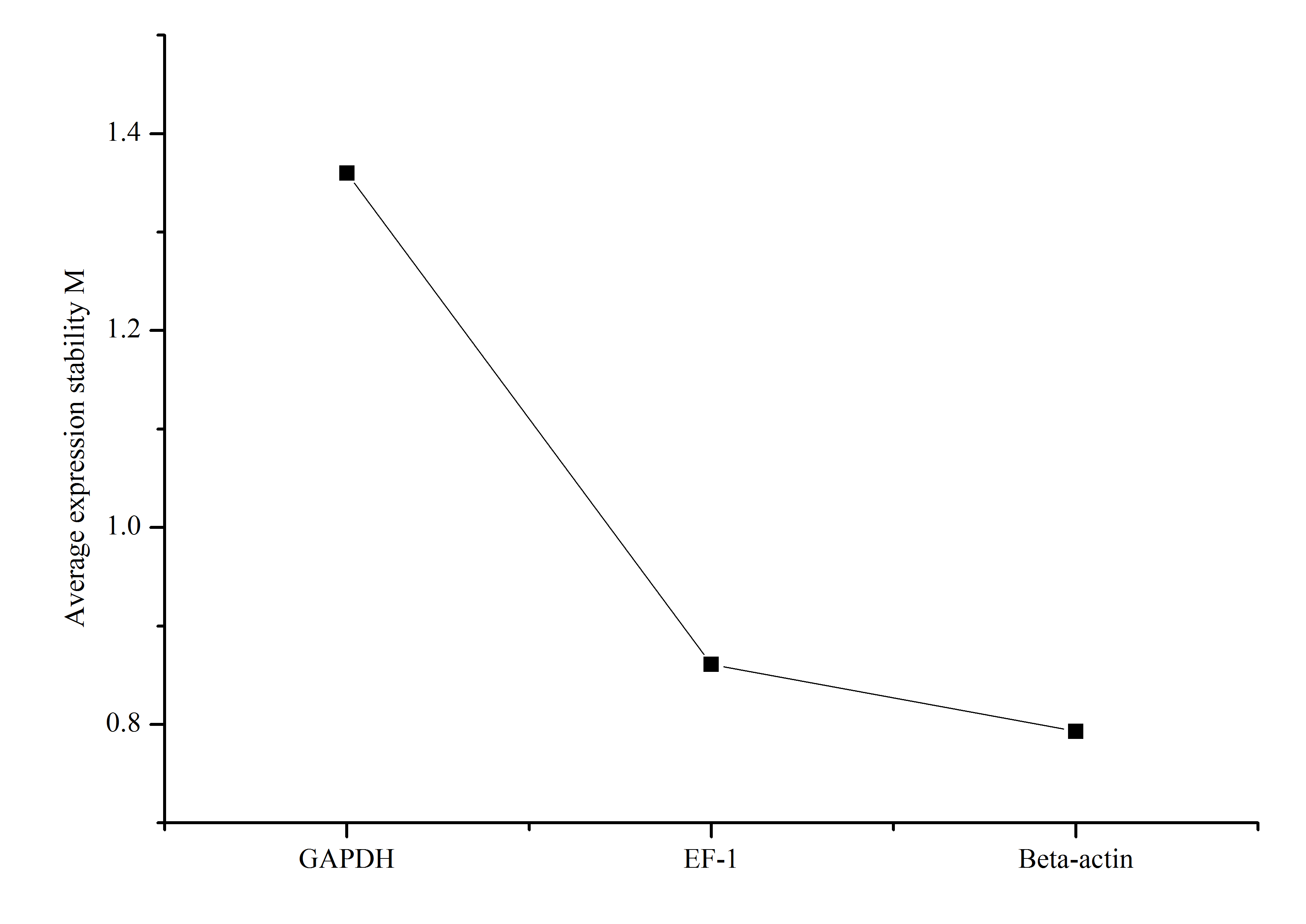

Supplement: Supplemental Information 5 — Gene expression stability of candidate reference genes in gonads was analyzed by the geNorm program. A lower value of average expression stability (M) indicates more stable expression. [file peerj-06-5389-s005.png]
